# SMHAT-1 mental health screening in Russian male professional soccer players: associations with age, competition level, and self-perceived team status

**DOI:** 10.3389/fspor.2026.1888491

**Published:** 2026-09-07

**Authors:** Eduard Bezuglov, Elizaveta Kapralova, Anna Berdnikova, Vladimir Khaitin, Alexander Rodionov, Mikhail Butovskiy, Zbigniew Waśkiewicz, Beatrisa Volel

**Affiliations:** 1 Department of Sports Medicine and Medical Rehabilitation, Sechenov First Moscow State Medical University, Ministry of Health of the Russia Federation, Moscow, Russia; 2 Department of Neurology, Institute of Professional Education, Sechenov First Moscow State Medical University of the Ministry of Health of the Russia Federation, Moscow, Russia; 3 Faculty of Postgraduate Education, Department of Physical Therapy and Sports Medicine, Pavlov First Saint Petersburg State Medical University, Saint Petersburg, Russia; 4 FC Locomotiv, Moscow, Russia; 5 Department of Rehabilitation and Sports Medicine, Kazan State Medical University of the Ministry of Health of the Russian Federation, Kazan, Russia; 6 Akademia Wychowania Fizycznego Imienia Jerzego Kukuczki w Katowicach, Katowice, Poland; 7 Department of Psychiatry and Psychosomatics, Sechenov First Moscow State Medical University of the Ministry of Health of the Russia Federation, Moscow, Russia; 8 Federal State Budgetary Scientific Institution Mental Health Research Center, Moscow, Russia

**Keywords:** APSQ, mental-health screening, professional soccer, psychological strain, SMHAT-1

## Abstract

**Introduction:**

Mental-health symptoms in professional athletes may impair performance, making timely screening important. This study examined Athlete Psychological Strain Questionnaire (APSQ; SMHAT-1 Step 1) scores and their associations with competition level, age, and self-rated team status in 170 male Russian soccer players.

**Methods:**

This cross–sectional study included 170 male Russian soccer players (mean age 21.1 ± 4.3 years). APSQ scores and their associations with competition level, age, and self–rated team status were assessed using ordinary least squares regression with HC3 robust standard errors.

**Results:**

Mean APSQ score was 17.8 ± 4.4, and 101/170 players screened positive at the ≥17 triage threshold (59.4%; 95% CI 51.9–66.5%). Age was positively associated with APSQ score (*b* = 0.28 points/year, 95% CI 0.07–0.48, *p* = 0.010). Self-rated team status showed an uncertain negative association (*b* = 0.28, 95% CI 0.63 to 0.07, *p* = 0.113). The overall four-level competition term was significant in the full model (*F*(3,164) = 3.37, *p* = 0.020); however, no planned contrast remained significant after false-discovery-rate correction, and the overall league test was not significant after exclusion of the four-player First League subgroup (*F*(2,161) = 1.52, *p* = 0.221).

**Discussion:**

These cross-sectional findings require cautious interpretation and confirmation in longitudinal, team-linked studies with further psychometric evaluation of the Russian adaptation.

## Introduction

Modern sport places high demands on athletes’ physical capabilities and mental performance. It is now well established that optimal psychological functioning is crucial for athletes’ professional success (1, 2). In this context, it is of practical importance to study the prevalence of psychopathological symptoms among professional athletes.

One of the most common methods of screening for these symptoms is the use of self-report questionnaires (3–5). The Sport Mental Health Assessment Tool 1 (SMHAT-1) was developed by experts from the International Olympic Committee in 2021 (6), which has since become a key instrument for screening the mental health of athletes. Several previous studies have applied the SMHAT-1 in diverse athlete populations (7–13). However, relatively few studies have applied the tool in professional soccer (14–16), and evidence remains limited regarding sport-specific symptom patterns and potential correlates in male professional players (17–19).

In elite team sports, psychological outcomes are shaped not only by competitive pressure but also by social dynamics within the team. An athlete's perceived influence, leadership role, or marginalization may contribute to psychological well-being or distress. Literature on social identity in sport suggests that perceived centrality or status can affect stress levels, coping mechanisms, and self-concept (20, 21). Age was also examined because the sample included youth and senior professionals at different career stages, which may involve different cumulative and transitional demands (2, 17–19). Self-perceived team status was therefore included as an exploratory psychosocial variable to examine whether perceived intragroup standing was associated with APSQ scores.

This study aimed to characterize APSQ scores and their associations with competition level, age, and self-rated team status. Domain-specific findings from the later SMHAT-1 forms were also summarized descriptively.

## Materials and methods

**Figure 1:**
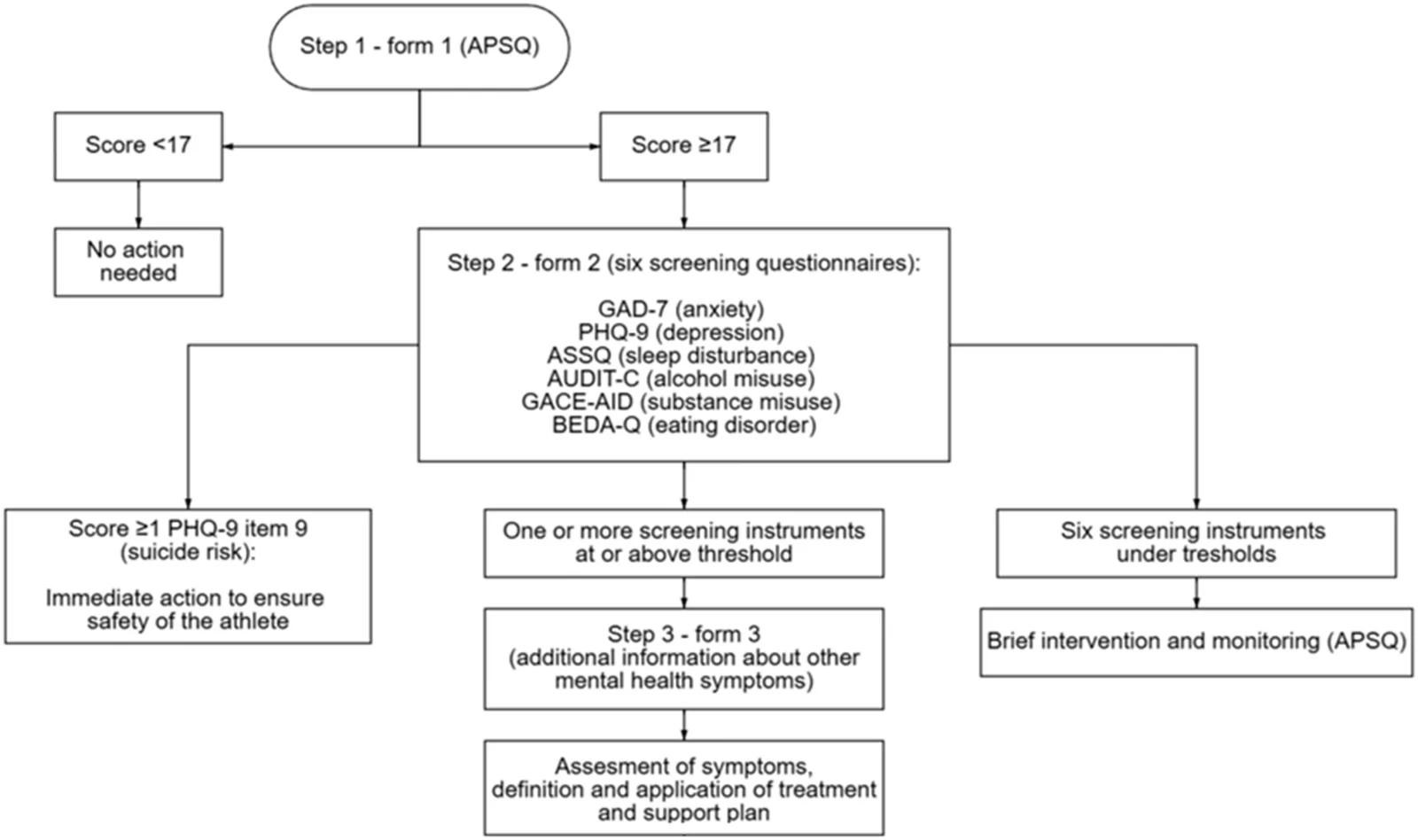
Author-created schematic of the SMHAT-1 assessment framework based on the algorithm described by Gouttebarge et al. (6). An APSQ score of ≥17 indicates administration of Form 2, and domain-specific Form 2 findings determine whether Form 3 is indicated. The arrows show the intended SMHAT-1 pathway and do not represent reconstructed participant-level transitions in this study.

This study included players from the first and youth squads of Russian Premier League clubs, as well as First and Second League teams. After receiving preliminary instructions on how to complete the questionnaire, participants completed the applicable Russian-language SMHAT-1 form(s) anonymously (Figure 1).

Written informed consent to take part in the study was provided by all participants. According to the IOC SMHAT-1 algorithm, an APSQ score below 17 concludes Step 1, whereas a score of 17 or higher indicates administration of Form 2; Form 2 findings determine whether Form 3 is indicated (Figure 1) (6). For confidentiality, each form used a separate anonymous identifier and no cross-form linkage key was retained. Individual transitions between forms could therefore not be reconstructed, and the three forms were analyzed as separate stage-specific datasets (Form 1, *n* = 170; Form 2, *n* = 55; Form 3, *n* = 51). Aggregate, non-identifiable findings were shared with team physicians. Individual transitions and follow-up actions could not be reconstructed from the research dataset.

The first part of the SMHAT-1 comprises the 10-item Athlete Psychological Strain Questionnaire (APSQ), which is sport-specific. Form 2 contains the GAD-7, PHQ-9, Athlete Sleep Screening Questionnaire sleep-difficulty score, AUDIT-C, CAGE-AID, and Brief Eating Disorder in Athletes Questionnaire. Form 3 contains the ASRS-v1.1 Part A, Mood Disorder Questionnaire, PC-PTSD-5, Problem Gambling Severity Index, and Prodromal Questionnaire-16. Each component instrument was scored separately according to the SMHAT-1 framework (6); no global Form 2 or Form 3 total was calculated.

Players with an APSQ score of 17 or higher were classified as APSQ-positive at triage, indicating the need for further assessment rather than a diagnosis.

Additionally, participants rated their perceived team status using an author-developed 1–10 item. A score of 1 represented one of the youngest players with an uncertain role regarding match participation, whereas 10 represented a recognized team leader. The item was used as an exploratory indicator of perceived standing and influence within the team and was not considered a validated multidimensional measure of team cohesion, leadership, role clarity, or social identity.

The inclusion criteria were male gender; age >=16 years; professional status [a valid contract and soccer as the primary source of income (22)], native-level proficiency in Russian; and an absence of time-loss injuries at the time of the study (23).

### Cross-cultural adaptation and psychometric evaluation

The SMHAT-1 was cross-culturally adapted for Russian-speaking users following established international guidelines (24). The Russian translation was reported to the IOC Mental Health Working Group by email on 31.10.2024. The adaptation process comprised five stages: (1) forward translation by two independent professional translators with backgrounds in sports medicine and psychology; (2) synthesis and reconciliation of the two translations into a single preliminary Russian version; (3) back-translation into English by a native English speaker who was blinded to the original version; (4) expert review by a panel of five specialists (two sports physicians, two clinical psychologists, and one professional soccer coach) who assessed content validity using a 4-point relevance scale; and (5) pilot testing on a convenience sample of 20 professional soccer players (mean age: 22.4 ± 3.8 years) from leagues not included in the main study sample. Following pilot testing, minor wording adjustments were made to improve clarity and cultural appropriateness.

Content Validity Index (CVI) at the item level (I-CVI) ranged from 0.80 to 1.00, with a Scale-level CVI (S-CVI/Average) of 0.92, indicating good content validity. Internal consistency for the Russian version of Form 1 (APSQ) was acceptable (Cronbach's *α* = 0.736). Domain-specific reliability estimates for Forms 2 and 3 are reported in Supplementary Tables S1 and S2. Although the Russian version underwent structured cross-cultural adaptation and preliminary content-validity assessment, confirmatory factor analysis, criterion validity, measurement invariance, and test-retest reliability require evaluation in larger independent samples before the version can be considered fully validated in this population.

### Design and privacy

Because this study concerned sensitive mental-health information, the survey was fully anonymized: no direct or indirect team identifiers were collected, and form-specific IDs did not provide a cross-form linkage key. Forms 1–3 were therefore analyzed as separate, unlinked stage-specific datasets rather than linked longitudinal observations. Multilevel modeling and empirical ICC estimation were not possible because team identifiers were unavailable.

### Statistical analysis

All statistical analyses were conducted in Python (version 3.11.13) using pandas 2.3.3, numpy 2.3.3, scipy 1.16.2, statsmodels 0.14.6, and matplotlib 3.10.8. Bootstrap analyses used 5,000 repetitions with random seed 20260728.

### Primary model (no dichotomization)

The APSQ was developed and psychometrically evaluated as a summed 10-item score (25), and a subsequent cut-off sensitivity study supported a score of ≥17 as indicating the need for further assessment (26). Accordingly, APSQ total score was analyzed as a continuous outcome using ordinary least squares (OLS) with HC3 heteroskedasticity-robust standard errors:

Competition level was encoded as a four-level factor (reference: Premier; other levels: Youth, First, Second). Regression coefficients are reported with 95% confidence intervals (CIs) and classical OLS degrees of freedom (*n* = 170; model *df* = 5; residual *df* = 164). Cluster-robust variance by league was not used because the number of clusters is small and true team-level clustering cannot be modeled without team identifiers.

### Planned contrasts and multiplicity

Three *a priori* contrasts estimated Youth minus Premier, First minus Premier, and Second minus Premier. Contrasts were evaluated using HC3 robust *t* tests with 164 residual degrees of freedom, and Benjamini–Hochberg FDR control (*q* = 0.05) was applied across exactly these three tests. Benjamini–Hochberg FDR control was selected to control the expected proportion of false discoveries within this related family of planned contrasts while retaining greater power than a family-wise error procedure.

### Secondary comparisons and effect sizes

For descriptive completeness, pairwise Welch's *t*-tests between competition levels were conducted and accompanied by Hedges’ *g* and Cliff's *δ* with 95% CIs (bootstrap for *δ*); Benjamini–Hochberg FDR correction was applied across all six pairwise comparisons.

### Precision and sensitivity to clustering

Estimation precision was emphasized over *post-hoc* power. For the Youth-minus-Premier planned contrast, the current 95% CI half-width was calculated as the t critical value with 164 residual degrees of freedom multiplied by the HC3 standard error. Because within-team dependence could not be modeled directly, a design-effect sensitivity grid was calculated over assumed ICCs of 0.05–0.25 and average team sizes of 10–25.

Model fit was summarized using *R*^2^, adjusted *R*^2^, and residual standard error, and the three league indicators were tested jointly using an HC3 robust Wald *F* test. Diagnostics included residual-vs.-fitted and Q-Q plots, Breusch-Pagan and White tests, Jarque–Bera test, Ramsey RESET, variance-inflation factors, leverage, externally studentized residuals, Cook's distance, and DFBETAs. Potential nonlinearity in age and team status was additionally examined by adding centered quadratic terms and testing them jointly using HC3 inference. Sensitivity analyses excluded the First League subgroup and observations with Cook's distance >4/n. APSQ-positive triage status (score >=17) was analyzed secondarily by logistic regression; because all four First League players were APSQ-positive, inferential logistic results were reported for the model excluding First League.

### Psychometrics and missing data

Cronbach's alpha was calculated separately for each coherent multi-item instrument. Form 2 and Form 3 domains were scored and reported separately, with screening-positive counts and Wilson 95% confidence intervals; global Form 2/Form 3 totals, global alpha values, and cross-domain item-total correlations were not calculated. Analyses used complete observations required for each model or scale, and denominators are reported explicitly.

## Results

The Form 1 analytic sample comprised 170 professional soccer players (mean age: 21.1 ± 4.3 years). Mean APSQ score was 17.82 ± 4.44, median: 18 [IQR: 14–21], and range: 10–29. Overall, 101/170 players were APSQ-positive (59.4%; Wilson 95% CI: 51.9%–66.5%). APSQ internal consistency was acceptable (Cronbach's alpha = 0.736). Descriptive statistics by competition level are shown in Table 1. The separate anonymous later-stage datasets contained 55 Form 2 and 51 Form 3 records and should not be interpreted as a trackable progression sequence.

**Table 1:** Descriptive statistics by competition level.

Competition level	*n*	APSQ total, mean ± SD	Median [IQR]	Range	APSQ >=17 *n* (%)	Age, mean ± SD	Team status, mean ± SD
Total	170	17.8 ± 4.44	18 [14–21]	10–29	101 (59.4)	21.06 ± 4.27	6.58 ± 2.39
Premier League	66	18.80 ± 4.92	18.5 [15.25–23]	10–29	45 (68.2)	24.00 ± 4.77	6.59 ± 2.78
Youth League	37	17.54 ± 3.88	18 [14–21]	10–24	22 (59.5)	17.76 ± 1.09	6.49 ± 1.91
First League	4	21.50 ± 1.91	22 [20.5–23]	19–23	4 (100.0)	24.00 ± 1.15	8.25 ± 0.50
Second League	63	16.71 ± 4.05	16 [14–19.5]	10–26	30 (47.6)	19.73 ± 2.65	6.51 ± 2.25

APSQ, Athlete Psychological Strain Questionnaire (SMHAT-1 Step 1; range: 10–50). APSQ > = 17 denotes positive triage rather than diagnosis. Team status was rated using an author-developed 1–10 item. First League findings are descriptive because *n* = 4.

In Form 2, positive-screen rates were 0.0% for GAD-7, 1.8% for PHQ-9, 1.8% for the sleep-difficulty score, 5.6% for AUDIT-C, 1.8% for CAGE-AID, and 41.8% for BEDA-Q. In Form 3, positive-screen rates were 2.0% for ASRS-v1.1, 3.9% for MDQ, 0.0% for PC-PTSD-5, 3.9% for PGSI problem gambling, and 9.8% for PQ-16. These findings describe the respondents to each unlinked stage-specific form and are not prevalence estimates for the complete Form 1 cohort (Supplementary Tables S1 and S2).

### Primary regression with HC3 standard errors

The primary OLS model explained 9.3% of APSQ-score variance (*R*^2^ = 0.093; adjusted *R*^2^ = 0.066; residual SE = 4.30). The overall competition-level term was significant in the full model [*F*(3,164) = 3.37, *p* = 0.020; delta *R*^2^ = 0.029 beyond age and team status]. Age was positively associated with APSQ score (*b* = 0.275 points/year, 95% CI: 0.066–0.485, *p* = 0.010), whereas the team-status estimate was negative but uncertain (*b* = −0.281, 95% CI: −0.629 to 0.067, *p* = 0.113). Relative to Premier League, the adjusted coefficients were 0.426 for Youth (p_FDR_ = 0.695), 3.163 for First (p_FDR_ = 0.070; exploratory), and −0.937 for Second League (p_FDR_ = 0.447); no planned contrast remained significant after FDR correction (Tables 2, 3).

**Table 2:** Primary OLS model with HC3 standard errors.

Term	*b*	SE (HC3)	95% CI	*t*	*p*	Partial *R*^2^
Intercept	14.05	2.28	[9.54, 18.56]	6.15	<0.001	0.188
Youth minus Premier	0.43	1.08	[−1.71, 2.57]	0.39	0.695	0.001
First minus Premier	3.16	1.38	[0.43, 5.89]	2.29	0.023	0.031
Second minus Premier	−0.94	0.90	[−2.71, 0.84]	−1.04	0.298	0.007
Age, per year	0.28	0.11	[0.07, 0.48]	2.59	0.010	0.039
Team status, per point	−0.28	0.18	[−0.63, 0.07]	−1.59	0.113	0.015

Outcome: continuous APSQ score. Premier League is the reference, so league coefficients are comparator minus Premier. *n* = 170; model *df* = 5; residual *df* = 164; *R*^2^ = 0.093; adjusted *R*^2^ = 0.066; residual SE = 4.30. Overall HC3 league test: *F*(3,164) = 3.37, *p* = 0.020. First League is exploratory (*n* = 4). Partial *R*^2^ values are approximate indices derived from robust t statistics; the intercept value is not interpreted.

**Table 3:** Planned adjusted contrasts.

Contrast	*b*	SE (HC3)	95% CI	*t*	*p*	*p* (FDR)
Youth minus Premier	0.43	1.08	[−1.71, 2.57]	0.39	0.695	0.695
First minus Premier	3.16	1.38	[0.43, 5.89]	2.29	0.023	0.070
Second minus Premier	−0.94	0.90	[−2.71, 0.84]	−1.04	0.298	0.447

Contrasts are comparator minus Premier and use HC3 robust *t* inference with 164 residual degrees of freedom. Benjamini–Hochberg FDR correction was applied across exactly three prespecified tests. The First League contrast is exploratory because *n* = 4.

**Figure 2:**
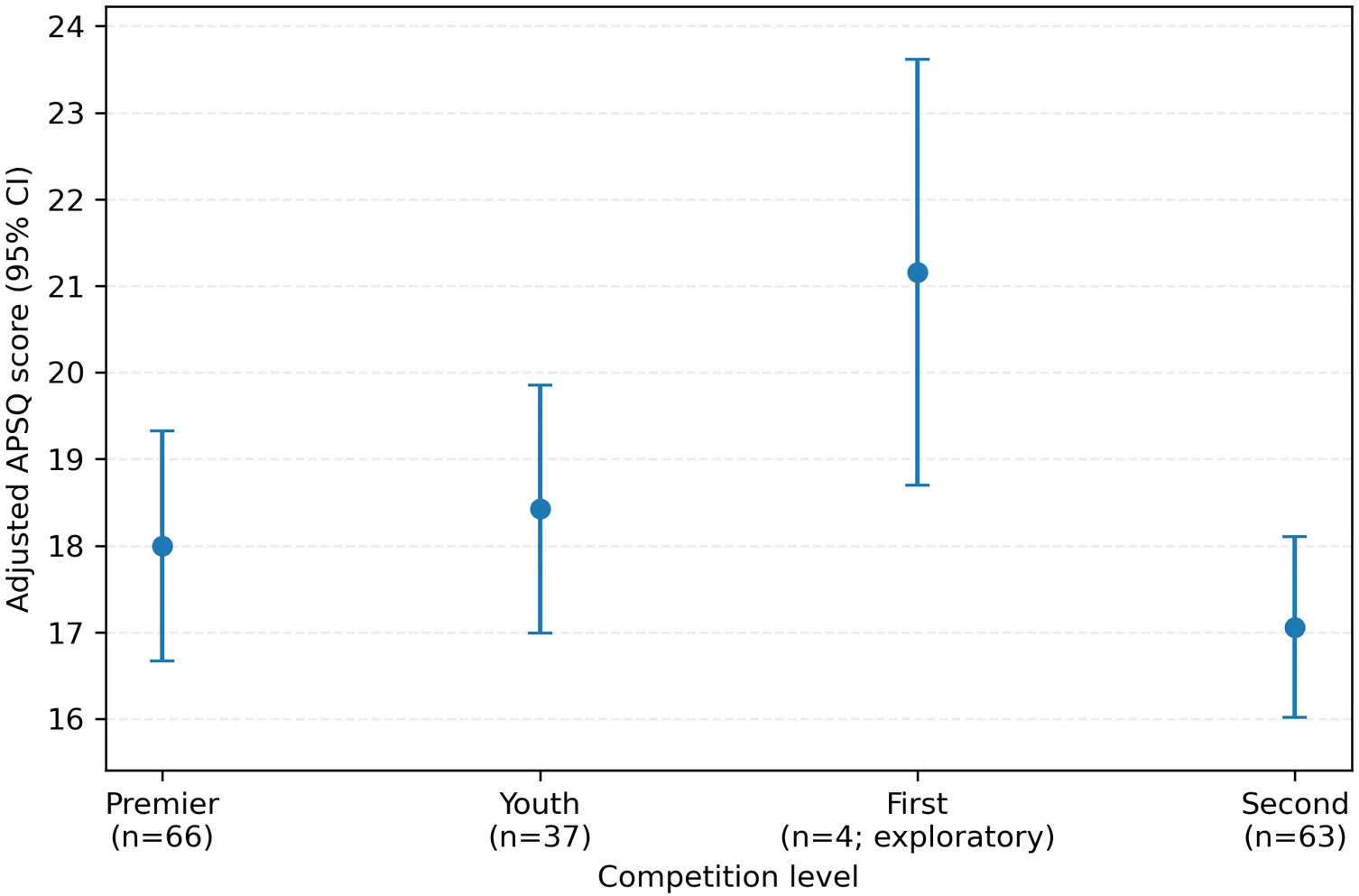
Adjusted mean APSQ score by competition level. Points are marginal means from the primary OLS model with age and self-rated team status held at their observed sample means; error bars are HC3 t-based 95% confidence intervals. First League is exploratory because *n* = 4.

Unadjusted effect sizes for the three planned comparisons are shown in Table 3A; the First League estimate is exploratory because *n* = 4. Full pairwise effect sizes for all six league comparisons are provided in Supplementary Table S3. Adjusted marginal means by competition level are shown in Figure 2.

**Table 3A:** Unadjusted effect sizes.

Contrast	Hedges’ *g*	95% CI	Cliff's *δ*	95% CI
Youth minus premier	−0.274	[−0.675, 0.127]	−0.148	[−0.371, 0.068]
First minus premier	0.553	[−0.449, 1.555]	0.367	[0.083, 0.629]
Second minus premier	−0.460	[−0.808, −0.112]	−0.255	[−0.443, −0.065]

Effect sizes are unadjusted and use the same comparator-minus-Premier direction as Table 3. Cliff's delta intervals used 5,000 bootstrap samples. First League estimates are exploratory because *n* = 4.

### Model diagnostics and sensitivity analyses

Diagnostic checks did not indicate major global model violations. The Breusch–Pagan and White tests did not indicate heteroskedasticity (*p* = 0.246 and *p* = 0.381, respectively), the Jarque–Bera test did not indicate substantial departure from residual normality (*p* = 0.214), and the Ramsey RESET test did not indicate functional-form misspecification (*p* = 0.607). Adding centred quadratic terms for age and team status did not improve model fit [joint HC3 *F*(2,162) = 1.40, *p* = 0.248]. Variance inflation factors ranged from 1.05 to 2.16. No externally studentized residual exceeded ∣3∣. Four observations exceeded the Cook's-distance threshold of 4/n, although the maximum Cook's distance was 0.044. Excluding these observations left the age and Youth- and Second-League coefficients materially similar; however, the team-status coefficient became slightly more negative and its confidence interval narrowly excluded zero (*b* = −0.316, 95% CI: −0.629 to −0.004; *p* = 0.047). This secondary association should therefore be interpreted cautiously because it showed some sensitivity to influential observations.

All four First League observations exceeded the leverage threshold, as expected when a category-specific coefficient is estimated from only four players. After excluding First League, the overall competition-level test was no longer significant [*F*(2,161) = 1.52, *p* = 0.221], while the age and team-status coefficients changed minimally. In the secondary APSQ-positive logistic model excluding First League because of complete separation, none of the predictors had a clearly estimated association. Detailed logistic results are reported in Supplementary Table S5, OLS sensitivity results in Supplementary Table S6, and graphical diagnostics in Supplementary Figure S1.

HC3 robust standard errors were retained for all regression inference regardless of the diagnostic-test results.

### Estimation precision and sensitivity to clustering

For the Youth-minus-Premier adjusted contrast, the HC3 standard error was 1.083, yielding a t-based 95% confidence-interval half-width of 2.14 APSQ points. Sample-size multipliers for alternative precision targets are shown in Table 4. Under simple inverse-square-root scaling, reducing the half-width from 2.14 to approximately 1.00 APSQ point would require about 4.58 times the current sample size. This calculation is an approximate planning illustration rather than a formal sample-size determination and does not account for team clustering.

**Table 4:** Precision targets for the youth-minus-premier contrast.

Target 95% CI half-width	Required multiplier (× current *n*)
1.50	2.03
1.25	2.93
1.00	4.58
0.75	8.13
0.50	18.31
0.40	28.59

Current 95% CI half-width = 2.14 APSQ points. Multipliers use approximate inverse-square-root scaling and are planning illustrations, not a formal clustered sample-size calculation. A half-width near 1 point would require about 4.6 times the current sample size.

Because team identifiers were not collected, within-team dependence could not be estimated or modeled directly. The hypothetical scenarios summarized in Table 5 produced standard-error inflation factors of approximately 1.20–2.65 and corresponding effective sample sizes of approximately 117–24. The full design-effect grid is provided in Supplementary Table S4. These assumed scenarios illustrate the potential loss of precision under within-team clustering but do not estimate the actual intraclass correlation and cannot replace observed team-level identifiers or multilevel modeling.

**Table 5:** Clustering sensitivity—benchmark scenarios.

Scenario	ICC	Assumed cluster size (m)	Deff	Neff	SE inflation
Optimistic	0.05	10	1.45	117.24	1.20
Moderate	0.10	18	2.70	62.96	1.64
Conservative	0.25	25	7.00	24.29	2.65

Team clusters and ICCs were not observed. The scenarios are hypothetical and do not estimate the true ICC or replace team-linked multilevel analysis. Design effect: Deff=1 + (*m* − 1) × ICC; Effective sample size: Neff = 170/Deff (Form 1 analytic *n*); SE inflation = Deff^0.5^.

## Discussion

The principal finding was that 101 of 170 players (59.4%) met the APSQ positive-triage threshold of ≥17. This does not establish a diagnosis of mental disorder but indicates psychological strain warranting further screening and, where appropriate, clinical assessment. Because Forms 1–3 used unlinked anonymous identifiers, participant-level progression to later stages could not be reconstructed. This study therefore adds evidence from a Russian male professional soccer sample to the growing football-specific literature applying the SMHAT-1 (14–16).

Previous international studies have examined heterogeneous athlete populations, generally evaluating mental health across different sports without distinguishing the specific prevalence patterns of psychopathological symptoms among elite athletes in particular sports. For example, Anderson et al. (7) surveyed a total of 1,066 athletes representing 51 different sports included in the programs of both the Summer and Winter Olympic and Paralympic Games. While this broad scope allows for a comprehensive assessment of the overall prevalence of mental health issues, it does not facilitate analysis of sport-specific correlations or associations. The SMHAT-1 questionnaire has been used to assess the mental health of athletes at various levels, from elite athletes to students at sports academies, in different countries. Similar studies have been conducted among Polish Olympic and Paralympic athletes (8, 9), Dutch Olympic and Paralympic athletes and coaching staff (10), Croatian adolescent athletes (11), and a multisport university program in Canada (12). Among larger studies focusing on athletes from a single sport, a notable example is a study of mental health disorders among Japanese Top League rugby players (13), which included 220 athletes.

The later-stage datasets showed substantial variation between screening domains. In Form 2, the BEDA-Q produced the highest positive-screen proportion (23/55; 41.8%), whereas positive screens on the other instruments were uncommon. In Form 3, the highest positive-screen proportion was observed for the PQ-16 (5/51; 9.8%). These findings should be interpreted cautiously because Forms 2 and 3 could not be linked to Form 1, several estimates had wide confidence intervals, and some domain-specific reliability coefficients were low. They therefore describe only the respondents to the respective forms and are not prevalence estimates for the complete Form 1 cohort.

Three studies are particularly noteworthy regarding the use of the SMHAT-1 among professional soccer players. In the study by Pillay et al. the mental health of 101 professional male soccer players from various countries (from first and second national leagues) was assessed, and a follow-up survey was conducted 12 months later (14). A similar study was carried out by Bilgoe et al. involving 74 professional female soccer players (15). Kilic et al. surveyed 149 male and 132 female Australian professional players using SMHAT-1 component instruments, including the APSQ (16).

In the present study, 101 of 170 players met the APSQ positive-triage threshold (59.4%; 95% CI: 51.9%–66.5%). This proportion was broadly comparable with the 52.5% reported by Pillay et al. and the 52% reported among male players by Kilic et al. (14, 16), and was higher than the 29.5% reported by Anderson et al. in a multisport Olympic and Paralympic cohort (7). These comparisons should nevertheless be interpreted cautiously because the studies differed in population characteristics, competitive context, timing of assessment, and implementation of the screening procedure. An APSQ-positive result indicates a need for further assessment and should not be interpreted as the prevalence of diagnosed psychopathology.

Studies have examined social identification in sport at personal, social, and collective levels (20, 21, 27). Social identity has been proposed as relevant to group behavior, social support and stress appraisal, leadership, and group-based emotional experiences in sport (20, 21). In addition, Pluhar et al. found higher levels of anxiety and depression among individual-sport than team-sport athletes (28), while the IOC consensus statement also noted that mental-health patterns may differ according to sporting context (2). Together, this literature provides a rationale for exploring perceived intrateam standing. However, the single status item used in the present study was not a measure of social identity itself and cannot represent the multidimensional identity processes described in previous research.

In the primary regression model, the adjusted association between self-perceived team status and APSQ score was negative but imprecisely estimated (*b* = −0.281, 95% CI: −0.629 to 0.067; *p* = 0.113). After excluding four observations exceeding the Cook's-distance threshold, the estimate became slightly more negative and its confidence interval narrowly excluded zero (*b* = −0.316, 95% CI: −0.629 to −0.004; *p* = 0.047). This sensitivity prevents firm conclusions about the relationship between perceived team status and psychological strain. Future studies should use validated multidimensional measures of social identification and related team processes.

We also compared Premier and Second League players, the two largest senior-league groups in the sample (*n* = 66 and *n* = 63, respectively). Premier League players were older on average than Second League players (24.00 ± 4.77 vs. 19.73 ± 2.65 years). In the unadjusted analysis, mean APSQ scores were higher among Premier League than Second League players (18.80 ± 4.92 vs. 16.71 ± 4.05; Welch *p* = 0.009; Hedges’ *g* = 0.46, 95% CI: 0.11–0.81). After adjustment for age and self-rated team status, however, the estimated difference for Second League relative to Premier League was −0.94 APSQ points (95% CI: −2.71 to 0.84; *p* = 0.298). Thus, the unadjusted difference was attenuated after adjustment and does not demonstrate a clear independent association between Premier-vs.-Second League status and APSQ score. It also does not justify attributing the difference to greater professional responsibility or psychological pressure, as these constructs were not directly measured. Median self-rated team status was 7 in both groups. More broadly, none of the planned league contrasts remained statistically significant after FDR correction, and the overall competition-level test was no longer significant after excluding the four First League players.

### Limitations

Several limitations should be considered when interpreting these findings. First, because this study addressed sensitive mental health information in a professional sport context, it was designed to be fully anonymous: no team identifiers were collected and individual IDs do not link across SMHAT-1 forms. While this approach is ethically appropriate, it prevented us from modeling players nested within teams and from estimating empirical intraclass correlations (ICCs). As a consequence, we could not fit multilevel models or use cluster-robust variance estimators; our primary regression instead relies on HC3 heteroskedasticity-robust standard errors. The design-effect sensitivity analysis (Table 5; Supplementary Table S4) shows that under the assumed ICC scenarios of 0.05–0.25 and average team sizes of 10–25 players, the effective sample size could be reduced to approximately 24–117 and standard errors inflated by about 1.2–2.6-fold. The true extent of within-team clustering, however, cannot be verified with the present data.

Second, the same anonymous design prevented us from linking individual responses across SMHAT-1 stages. We could not determine which APSQ-positive athletes also exceeded thresholds on specific secondary screeners, nor could we estimate the prevalence of particular syndromes (e.g., anxiety, depression, substance use disorders) within this cohort. Domain-specific findings from the later-stage forms can be described among their respondents, but cross-form concordance, conditional progression, and full-cohort domain prevalence cannot be estimated.

Third, although the overall Form 1 sample size was moderate (*n* = 170), some subgroups were very small—most notably the First League (*n* = 4). Estimates and effect sizes for these strata, including the corresponding contrasts in Table 3A and Supplementary Table S3, are therefore exploratory and should be interpreted with caution. All four First League observations had high leverage, and the overall competition-level test was no longer statistically significant after this subgroup was excluded. Consequently, the full-model league result should not be interpreted as robust evidence of competition-level differences.

Fourth, self-perceived team status was measured using a single 10-point item. This pragmatic approach facilitated data collection in an applied setting, but likely underrepresents the multidimensional nature of social identity and intra-team roles. Measurement imprecision in this construct may have contributed to the wide confidence intervals and our inability to detect small associations between perceived status and APSQ scores.

Fifth, the primary model explained only a modest proportion of the variance in APSQ scores (adjusted *R*^2^ = 0.066). Age, competition level, and self-rated team status therefore capture only a small subset of the individual, interpersonal, and organizational factors potentially associated with psychological strain.

Finally, the sample consisted of male professional soccer players from Russian Premier, First, Second and Youth leagues. The findings may not generalize to female players, athletes from other countries or sports, or non-professional levels of competition.

Previous studies on the mental health of soccer players have been limited by a lack of standardized approaches to questionnaire selection and symptom assessment (17–19), which hinders the comparability of results across different research groups. The SMHAT-1 provides a structured athlete-specific pathway combining initial psychological-strain triage with domain-specific follow-up screening (6). Standardized athlete-specific screening may improve comparability across studies, but future work should combine SMHAT-1 with longitudinal linkage, team identifiers, multidimensional measures of team context, and formal clinical assessment where indicated (2, 6, 20, 21, 27).

## Conclusions

In this cross-sectional cohort of Russian male professional soccer players, age was positively associated with APSQ score, whereas the association with self-rated team status was uncertain. Competition-level findings were not robust to exclusion of the four-player First League subgroup, and no planned league contrast remained significant after FDR correction. Forms 1–3 could not be linked at participant level, so later-stage findings are descriptive of their respective respondents. Longitudinal, team-linked studies using multidimensional psychosocial measures and further psychometric evaluation of the Russian adaptation are required.

## Data Availability

The raw data supporting the conclusions of this article will be made available by the authors, without undue reservation.
