# Supplementary material for: SMHAT-1 mental health screening in Russian male professional soccer players: associations with age, competition level, and self-perceived team status
Source: Front Sports Act Living. 2026 Sep 7;8:1888491. doi: 10.3389/fspor.2026.1888491 (PMC13591499; doi:10.3389/fspor.2026.1888491)
Supplement: Supplementary file 1 [file Supplementaryfile1.docx]

Supplementary Table S1. Form 2 domain-specific results

| Scale | n | Mean ± SD | Median [IQR] | Range | Positive n (%) | Wilson 95% CI | alpha |
| --- | --- | --- | --- | --- | --- | --- | --- |
| GAD-7 | 55 | 2.44 ± 2.26 | 2 [1-3] | 0-9 | 0 (0.0) | [0.0, 6.5] | 0.726 |
| PHQ-9 | 55 | 2.95 ± 2.10 | 2 [2-3.5] | 0-12 | 1 (1.8) | [0.3, 9.6] | 0.555 |
| ASSQ sleep score | 55 | 3.89 ± 1.90 | 4 [3-5] | 1-12 | 1 (1.8) | [0.3, 9.6] | 0.585 |
| AUDIT-C | 54 | 0.87 ± 1.51 | 0 [0-1] | 0-7 | 3 (5.6) | [1.9, 15.1] | 0.648 |
| CAGE-AID | 55 | 0.15 ± 0.59 | 0 [0-0] | 0-4 | 1 (1.8) | [0.3, 9.6] | 0.793 |
| BEDA-Q | 55 | 3.22 ± 2.43 | 3 [1-5] | 0-9 | 23 (41.8) | [29.7, 55.0] | 0.336 |

Note. Positive rules: GAD-7 >=10; PHQ-9 >=10; ASSQ sleep-difficulty score >=8; AUDIT-C >=4 for men; CAGE-AID >=2; BEDA-Q >=4 (IOC-proposed cutoff). One respondent endorsed the PHQ-9 self-harm item. Reliability estimates are exploratory in this small stage-specific dataset; no global Form 2 alpha or total was calculated.

Supplementary Table S2. Form 3 domain-specific results

| Scale | n | Mean ± SD | Median [IQR] | Range | Positive n (%) | Wilson 95% CI | alpha/ KR20 |
| --- | --- | --- | --- | --- | --- | --- | --- |
| ASRS-v1.1 | 51 | 0.57 ± 1.02 | 0 [0-1] | 0-4 | 1 (2.0) | [0.3, 10.3] | 0.622 |
| MDQ | 51 | 4.04 ± 2.88 | 4 [1.5-5.5] | 0-12 | 2 (3.9) | [1.1, 13.2] | 0.791 |
| PC-PTSD-5 | 51 | 0.06 ± 0.31 | 0 [0-0] | 0-2 | 0 (0.0) | [0.0, 7.0] | 0.385* |
| PGSI | 51 | 0.86 ± 2.11 | 0 [0-0] | 0-9 | 2 (3.9) | [1.1, 13.2] | 0.877 |
| PQ-16 | 51 | 2.53 ± 2.48 | 2 [1-3] | 0-14 | 5 (9.8) | [4.3, 21.0] | 0.747 |

Note. Positive rules: ASRS-v1.1 at least four positive Part A items; MDQ >=7 symptoms plus co-occurrence and impairment; PC-PTSD-5 trauma exposure plus symptom score >=4; PGSI >=8; PQ-16 >=6 endorsed symptoms. *PC-PTSD-5 reliability was calculated among seven trauma-exposed respondents and is highly unstable. No global Form 3 alpha or total was calculated.

Supplementary Table S3. Pairwise league comparisons on APSQ total (Form 1): Welch tests, Hedges’ g, and Cliff’s δ

| Contrast | n1 | n2 | Mean1 ± SD | Mean2 ± SD | Welch t | Welch df | p | p (FDR) | Hedges’ g | 95% CI (g) | Cliff’s δ | 95% CI (δ) |
| --- | --- | --- | --- | --- | --- | --- | --- | --- | --- | --- | --- | --- |
| Premier vs Youth | 66 | 37 | 18.803 ± 4.915 | 17.541 ± 3.884 | 1.435 | 89.637 | 0.155 | 0.186 | 0.274 | [−0.127, 0.675] | 0.148 | [−0.075, 0.370] |
| Premier vs First | 66 | 4 | 18.803 ± 4.915 | 21.500 ± 1.915 | −2.381 | 5.832 | 0.056 | 0.084 | −0.553 | [−1.555, 0.449] | −0.367 | [−0.621, −0.087] |
| Premier vs Second | 66 | 63 | 18.803 ± 4.915 | 16.714 ± 4.046 | 2.640 | 124.343 | 0.009 | 0.026 | 0.460 | [0.112, 0.808] | 0.255 | [0.065, 0.443] |
| Youth vs First | 37 | 4 | 17.541 ± 3.884 | 21.500 ± 1.915 | −3.440 | 6.161 | 0.013 | 0.026 | −1.030 | [−2.067, 0.007] | −0.622 | [−0.919, −0.257] |
| Youth vs Second | 37 | 63 | 17.541 ± 3.884 | 16.714 ± 4.046 | 1.011 | 78.082 | 0.315 | 0.315 | 0.206 | [−0.198, 0.610] | 0.135 | [−0.102, 0.361] |
| First vs Second | 4 | 63 | 21.500 ± 1.915 | 16.714 ± 4.046 | 4.412 | 4.923 | 0.007 | 0.026 | 1.191 | [0.171, 2.210] | 0.698 | [0.464, 0.889] |

Notes. Welch tests (unequal variances). p (FDR) = Benjamini–Hochberg adjustment across the six pairwise tests. Hedges’ g is bias-corrected; 95% CIs via Hedges–Olkin normal approximation. Cliff’s δ 95% CIs via bootstrap percentiles (5,000 resamples). Small n in First (n=4) warrants caution. For each row, mean differences and effect sizes are calculated as the first listed group minus the second listed group.

Supplementary Table S4. Design-effect sensitivity (effective sample size and SE inflation)

| **ICC** | Assumed cluster size (m) | **Deff** | **Neff** | **SE inflation** |
| --- | --- | --- | --- | --- |
| 0.05 | 10 | 1.45 | 117.241 | 1.204 |
| 0.05 | 12 | 1.55 | 109.677 | 1.245 |
| 0.05 | 15 | 1.70 | 100.000 | 1.304 |
| 0.05 | 18 | 1.85 | 91.892 | 1.360 |
| 0.05 | 20 | 1.95 | 87.179 | 1.396 |
| 0.05 | 22 | 2.05 | 82.927 | 1.432 |
| 0.05 | 25 | 2.20 | 77.273 | 1.483 |
| 0.10 | 10 | 1.90 | 89.474 | 1.378 |
| 0.10 | 12 | 2.10 | 80.952 | 1.449 |
| 0.10 | 15 | 2.40 | 70.833 | 1.549 |
| 0.10 | 18 | 2.70 | 62.963 | 1.643 |
| 0.10 | 20 | 2.90 | 58.621 | 1.703 |
| 0.10 | 22 | 3.10 | 54.839 | 1.761 |
| 0.10 | 25 | 3.40 | 50.000 | 1.844 |
| 0.15 | 10 | 2.35 | 72.340 | 1.533 |
| 0.15 | 12 | 2.65 | 64.151 | 1.628 |
| 0.15 | 15 | 3.10 | 54.839 | 1.761 |
| 0.15 | 18 | 3.55 | 47.887 | 1.884 |
| 0.15 | 20 | 3.85 | 44.156 | 1.962 |
| 0.15 | 22 | 4.15 | 40.964 | 2.037 |
| 0.15 | 25 | 4.60 | 36.957 | 2.145 |
| 0.20 | 10 | 2.80 | 60.714 | 1.673 |
| 0.20 | 12 | 3.20 | 53.125 | 1.789 |
| 0.20 | 15 | 3.80 | 44.737 | 1.949 |
| 0.20 | 18 | 4.40 | 38.636 | 2.098 |
| 0.20 | 20 | 4.80 | 35.417 | 2.191 |
| 0.20 | 22 | 5.20 | 32.692 | 2.280 |
| 0.20 | 25 | 5.80 | 29.310 | 2.408 |
| 0.25 | 10 | 3.25 | 52.308 | 1.803 |
| 0.25 | 12 | 3.75 | 45.333 | 1.936 |
| 0.25 | 15 | 4.50 | 37.778 | 2.121 |
| 0.25 | 18 | 5.25 | 32.381 | 2.291 |
| 0.25 | 20 | 5.75 | 29.565 | 2.398 |
| 0.25 | 22 | 6.25 | 27.200 | 2.500 |
| 0.25 | 25 | 7.00 | 24.286 | 2.646 |

Supplementary Table S5. APSQ-positive logistic sensitivity excluding First League

| Predictor | OR | 95% CI | p |
| --- | --- | --- | --- |
| Youth vs Premier | 1.26 | [0.43, 3.69] | 0.675 |
| Second vs Premier | 0.64 | [0.27, 1.50] | 0.302 |
| Age, per year | 1.11 | [0.99, 1.24] | 0.073 |
| Team status, per point | 0.89 | [0.76, 1.05] | 0.160 |

Note. n = 166; 97 APSQ-positive events. First League was excluded because all four First League players were APSQ-positive, causing complete separation in ordinary logistic regression. HC3 robust standard errors were used.

Supplementary Table S6. OLS sensitivity analyses

| Analysis / term | b | 95% CI | p |
| --- | --- | --- | --- |
| Exclude First: Youth minus Premier | 0.443 | [-1.699, 2.584] | 0.684 |
| Exclude First: Second minus Premier | -0.926 | [-2.700, 0.849] | 0.304 |
| Exclude First: Age | 0.278 | [0.068, 0.488] | 0.010 |
| Exclude First: Team status | -0.285 | [-0.633, 0.064] | 0.109 |
| Exclude Cook >4/n: Youth minus Premier | 0.589 | [-1.473, 2.652] | 0.573 |
| Exclude Cook >4/n: First minus Premier | 4.327 | [2.337, 6.317] | <0.001 |
| Exclude Cook >4/n: Second minus Premier | -0.778 | [-2.480, 0.925] | 0.369 |
| Exclude Cook >4/n: Age | 0.277 | [0.074, 0.481] | 0.008 |
| Exclude Cook >4/n: Team status | -0.316 | [-0.629, -0.004] | 0.047 |

Note. The no-First model included n = 166 and yielded F(2,161) = 1.52, p = 0.221 for the overall league term. The influence sensitivity excluded four observations with Cook’s distance >4/n. These exclusions are sensitivity analyses, not recommendations to discard observations.

Supplementary Figure S1. Diagnostics for the primary APSQ OLS model: (A) residuals versus fitted values; (B) Q-Q plot; (C) Cook’s distance with the 4/n reference line; and (D) leverage versus externally studentized residuals with reference lines at 2p/n and ±3, respectively
